# A new two-phase dimeticone pediculicide shows high efficacy in a comparative bioassay

**DOI:** 10.1186/1471-5945-9-12

**Published:** 2009-12-14

**Authors:** Jorg Heukelbach, André Asenov, Oliver Liesenfeld, Ali Mirmohammadsadegh, Fabíola A Oliveira

**Affiliations:** 1 Department of Community Health, School of Medicine, Federal University of Ceará, Rua Prof. Costa Mendes 1608, 5. andar, Fortaleza CE 60430-140, Brazil; 2 Anton Breinl Centre for Tropical Medicine and Public Health; School of Public Health, Tropical Medicine and Rehabilitation Sciences, James Cook University, Townsville, Australia; 3 Department of Hygiene and Microbiology, Charité Medical School, Campus Benjamin Franklin, Berlin, Germany; 4 Department of Dermatology, University of Düsseldorf, Düsseldorf, Germany

## Abstract

**Background:**

Dimeticones kill head lice by physical means. Here we assessed in a comparative bioassay the *ex vivo *efficacy of "NYDA^® ^sensitiv", a new two-phase dimeticone-based pediculicide similar to a product established on the market, but without fragrances.

**Methods:**

We compared efficacy of the new product to a positive dimeticone control group, a sample of four other insecticidal and natural head lice products marketed in Germany, and an untreated control. In a bioassay, lice were exposed *ex vivo *to products and examined for activity for up to 24 hours, following a standard protocol.

**Results:**

After 6 and 24 hours, 13.7 and 88.5% of untreated control lice did not show major vital signs. In contrast, no lice showed major vital signs 5 minutes after treatment with the new product or the control dimeticone group (NYDA^®^). This effect persisted at all observation points (100% efficacy). Efficacy of 0.5% permethrin (Infectopedicul^®^) ranged between 76 and 96% in evaluations between 5 min and 6 hours. All lice treated with a coconut-based compound (mosquito^® ^Läuseshampoo) did not show major vital signs after 5 min, but mortality was only 58% after one hour. Pyrethrum extract (Goldgeist^® ^forte) showed an efficacy of 22 - 52% between 5 min and 3 hours after treatment; after 6 hours, 76% of lice were judged dead. An oxyphthirine^®^-based compound (Liberalice DUO LP-PRO^®^) killed 22 - 54% of lice in the first 6 hours.

**Conclusions:**

The two-phase dimeticone compound NYDA^® ^sensitiv is highly efficacious. The removal of fragrances as compared to an established dimeticone product did not affect *in vitro *efficacy.

## Background

Head lice infestations are widespread and occur in all socio-economic strata in low, middle and high-income countries [1-3]. Anecdotal evidence suggests an increasing prevalence of head lice in many countries. In addition, as a result of the extensive use of neurotoxic insecticides, such as permethrin and malathion, reports of resistances to these pediculicides have increased in the recent years [4-8].

However, many over-the-counter head lice products are still not tested adequately for their efficacy - neither before nor after licensing - and, unfortunately, lack efficacy. For example, we recently reported that only one of six plant-based head lice products marketed in Australia showed a considerable degree of efficacy against adult head lice [9]. Thus, there is a clear need for standardized assessment of alternative head lice treatments. In fact, there is now a trend for a more evidence-based approach, and there have been several promising reports of clinical trials and bioassays testing new topical head lice compounds [10-19].

The trends for product development in some countries have changed from neurotoxic insecticides to products containing substances acting by physical means [20]. These compounds, such as silicones, are of particular interest for head lice control: they are considered non-toxic to humans, and due to their mode of action development of resistance is not expected [12,20-22]. The silicone oil dimeticone coats the louse surface and enters the respiratory tract, thereby blocking spiracles and tracheae [20,22]. Böckeler & Richling (2008) showed that a two-phase product containing a mixture of two dimeticones of different viscosity (NYDA^®^) was capable of entering the tracheal system, subsequently asphyxiating lice [22]. We have previously shown in a clinical trial and in an *ex vivo *study that this two-phase dimeticone (marketed in Germany, Austria, the UK, France, Denmark, Finland, Greece, The Netherlands, Turkey and several other countries) is highly efficacious against head lice [12,13]. Here we report efficacy of a similar formulation, but without fragrances, and compare it to several other products.

## Methods

The efficacy of "NYDA^® ^sensitiv" was assessed in a bioassay, using adult head lice collected from heavily infested individuals. We compared efficacy to a positive control group, a sample of four other insecticidal and natural head lice products on the German market, and an untreated control group. The selection of products was based on the aim of the study: to prove similar efficacy of a new dimeticone formula, as compared to an established two-phase dimeticone; and to compare these dimeticone pediculicides with products based on other modes of action (neurotoxic or plant-based), with a considerable market share in Germany. Details of the six products tested are depicted in Table 1.

**Table 1:** Details of products tested against head lice.

	Ingredients	Presentation	Producer
NYDA^® ^sensitiv	92% dimeticone, medium-chain triglycerides, jojoba wax	Solution in plastic bottle	G. Pohl-Boskamp GmbH o KG, Hohenlockstedt (Germany)
NYDA^®^	92% dimeticone, medium-chain triglycerides, jojoba wax, fragrances	Solution in glass bottle	G. Pohl-Boskamp GmbH o KG, Hohenlockstedt (Germany)
Liberalice DUO LP-PRO^®^	Oxyphthirine^® ^(triglycerides and lipid esters)*	Lotion in plastic bottle	Duhot S.A., Limal (Belgium)
Infectopedicul^®^	0.5% permethrin, ethanol (39 vol. %), 2-propanol, purified water, propylene glycol, sodium dihydrogen phosphate	Solution in glass bottle	InfectoPharm Arzneimittel und Consilium GmbH, Heppenheim (Germany)
Goldgeist^® ^forte	Pyrethrum extract (0,3%), piperonyl butoxide, chlorocresol, diethylene glycol	Solution in glass bottle	Eduard Gerlach GmbH, Lübbecke (Germany)
mosquito^® ^Läuseshampoo	Soybean oil, coconut oil derivatives, purified water, sodium laureth sulphate, cocamidopropyl betaine, glycine soya, hydrolysed collagen, glycosphingolipids, geranium	Plastic bottle/shampoo	Wepa Apothekenbedarf GmbH o. KG, Hillscheid (Germany)

* no further information available

Product names as marketed in Germany.

The new product differs from the established dimeticone product by the absence of fragrances. As a consequence, concentrations of dimeticones are slightly changed. The compound without fragrances contains a mixture of two dimeticones of different viscosity, in a total concentration of 92.4%, as compared to 92.0% in the product with fragrances. Fragrances used in NYDA^® ^are α-terpineol and *Eucalyptus citriodora *oil. The remaining difference in concentration between both dimeticone formulations was substituted by middle-chain triglycerides, which is another ingredient of both products.

### Head lice used

Adult head lice (*Pediculus capitis*) were obtained by dry combing with a fine toothed comb, from children living in a community in the city of Fortaleza (northeast Brazil), where head lice are endemic. Children had not treated their infestations with topical compounds or taken any antibiotics/antiparasitic drugs in the previous four weeks. We examined lice for activity and physical integrity and used fully active adult insects within 60 min after collection. Female and male lice were used.

### Pediculicidal bioassay

A previously published standardized method for *ex vivo *assessment of efficacy of pediculicidal products was used [9,13]: Lice attached to hair strands were immersed completely in undiluted products for 3 min and then placed with hairs onto Whatman filter paper in 5 cm Petri dishes. To prevent lice from desiccation, the filter paper was moistened by 200 μl tap water. After placing lice on filter paper, obvious pools of the products were wiped from the lice by a jeweller's forceps directed under a dissecting microscope. Lice were washed after 20 min by immersing the strands into water for one minute. Hair strands and lice were agitated using a jeweller's forceps, and then placed on new Petri dishes with filter paper. Lice of the untreated control group were washed after 20 min without any further treatment.

The number of parasites tested was 50 in each treatment and 131 in the control group. Lice were tested in batches of nine to eleven insects, and results were then pooled.

The lice were examined for activity under a dissecting microscope after 5, 10, 20, 30, 60, 120 and 180 min, as well as after 6 hours. A final assessment was made after 24 hours - however, a high mortality in the control group was expected, as lice desiccate without regular feeding.

Pre-defined criteria for evaluation of survival of lice were used, based on activity, ataxic signs, ability to stay on hair, as well as gut and leg movements. As the exact time point of death of an insect is difficult to define, we defined strict criteria for the determination of "mortality": Lice were judged as "dead" if there were no vital signs or minor vital signs present (merely internal gut movements, movements of antennae, minimal leg movements with or without stimulation by a forceps). Lice showing major vital signs such as inability to walk in a progressive fashion or no righting reflex when rolled onto the back - considered as dead in many other bioassays [9] - were judged as alive.

A single observer performed all examinations to prevent inter-observer variation. Due to differences in color and odor of products, complete blinding was impossible. All lice were maintained at 27-29°C and not fed during testing.

### Statistical analysis

Data were entered using Excel spreadsheets and checked for entry-related errors. Binomial confidence intervals of mortality rates were calculated using STATA^® ^software version 8.2 (Stata corporation, College Station, USA). The significance of the difference of relative frequencies was compared by applying Fisher's exact test.

### Ethical aspects

The study was done in collaboration with local community leaders and approved by the Ethical Review Board of the Federal University of Ceará (Fortaleza, Brazil). Prior to combing, study objectives were explained, and informed written consent was obtained from children and their guardians. All children and their families involved were treated against head lice and other parasitic diseases with oral ivermectin (Revectina^®^; 200 μg/kg body weight, repeated after 10 days), or in the case of contra-indications, with a 1% permethrin-based lotion available over the counter in Brazil (Kwell^®^). In Brazil, oral ivermectin is registered for treatment of pediculosis and has been shown to be effective against a variety of parasitic diseases [23].

## Results

**Figure 1:**
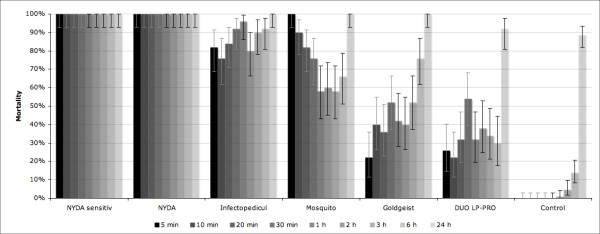
**Comparative efficacy (mortality) of head lice products at different points in time (n = 50 in test groups; n = 131 in control group)**. Vertical bars indicate binomial 95% confidence intervals.

Figure 1 details efficacies of the six tested products at different points in time, as compared to the untreated control group. All lice treated with both two-phase dimeticones showed no major vital signs after 5 min, and this effect persisted at all points in time (100% efficacy). Efficacy of 0.5% permethrin (Infectopedicul^®^) ranged between 76 and 96% in evaluations between 5 min and 6 hours. All lice treated with mosquito^® ^did not show major vital signs after 5 min, but recovered, and mortality was only 58% after one hour. Goldgeist^® ^forte showed an efficacy of 22 - 52% between 5 min and three hours after treatment. After six hours, 76% of lice were dead. However, in this group after 10 min 48%, and after 3 hours 36% of lice were fully active. Liberalice DUO LP-PRO^® ^killed 22 - 54% of lice in the first 6 hours. Independent of the product applied, after 24 hours all lice were dead with exception of lice treated with Liberalice DUO LP-PRO^® ^(92%, Figure 1).

In the untreated control group, all lice survived for up to one hour. After six hours 13.7% of lice were dead, and after 24 hours 88.5% (Figure 1).

The two-phase dimeticones performed significantly better than Infectopedicul^® ^at observations after 5 min (p = 0.003), 10 min (p < 0.001), 20 min (p = 0.006) and 2 h (p = 0.001); better than mosquito^® ^at observations after 20 min (p = 0.003), 30 min, 1 h, 2 h, 3 h and 6 h (all p < 0.001); and better than Liberalice DUO LP-PRO^® ^and Goldgeist^® ^forte at all observations in the first six hours after exposure to products (all p < 0.001). At other observation points, differences between products tested were not statistically significant.

## Discussion

Our data show that a new dimeticone-based head lice product without fragrances is highly efficacious in a standardized pediculicidal bioassay. The removal of α-terpineol and *Eucalyptus citriodora *did not affect efficacy, as compared to the established two-phase dimeticone product NYDA^®^.

Several recent studies have shown the efficacy of products containing silicone oils. In clinical trials, preparations based on silicones have shown a high efficacy as compared to neurotoxic insecticides [11,12,14,24,25]. In a recent comparative clinical trial, efficacy of two-phase dimeticone was 97% after 9 days, as compared to 68% efficacy of 1% permethrin [12]. Considering the similar *ex vivo *efficacy of both two-phase dimeticones used in the present study, it can be assumed that clinical efficacy of the product without fragrances will be similar. In addition, an *in vitro *ovicidal assay has shown that the two-phase dimeticone reveals almost 100% efficacy against head lice eggs [26].

In a previous comparative bioassay we observed 100% efficacy of NYDA^® ^and 1% malathion (Prioderm^®^), compared to 90% efficacy of 1% permethrin creme rinse (Lyclear^®^) [13]. Lice treated with 4% dimeticone/96% cyclomethicone (Hedrin^®^) showed a similar efficacy to two-phase dimeticone in the first three hours after exposure, but a considerable number of lice recovered after 6 hours [13].

In the present study, an alcoholic solution of 0.5% permethrin showed an efficacy ranging between 72 and 96% in the first six hours after exposure. Similar results applying an identical methodology were found with permethrin-based products from Australia, the UK and Brazil [9,13,27]. The pyrethrum extract Goldgeist^® ^forte showed a low efficacy in our study.

Liberalice DUO LP-PRO^® ^contains a compound called oxyphthirine^®^, which is based on triglycerides and lipid esters. Lice are claimed to be killed by asphyxiation, but no adequate *in vitro *or clinical data are published confirming this assumption. In our assay, efficacy of this product was low, indicating that a single topical application, as recommended by the producer, is not sufficient to eradicate lice. Resistance is an unlikely cause for the low efficacy, as the assays were performed in Brazil where the product is not commercialized yet and thus lice were most likely naïve regarding exposure to oxyphthirine^®^.

A considerable number of lice exposed to the plant-based product mosquito^® ^recovered of sham death after some time. Resistance may develop also against plant extracts, but similar to permethrin we cannot conclude on resistance patterns. In fact, death of insects is difficult to define, and lice in particular have the ability to shut down their metabolism and go into stasis; they may later recover from an apparently morbid state [28,29]. Thus, in the evaluation of head lice treatments, the end-point "death" can only be determined by microscopy and by observing the insects for a prolonged period, as done in our study. Some authors have highlighted the need for an extended observation period of at least 6 hours to confirm death [13,27,28,30]. From the design of above cited studies and data presented in this study, it cannot be concluded if resistance is the cause for incomplete efficacy of pyrethroids and plant products. This should be the focus of future studies. Resistance of the Brazilian lice tested has not yet been described.

Since our study was performed, another product (50% isopropyl myristate, a fatty acid ester, and 50% cylcomethicone) claimed killing lice by physical means has entered the German market. Clinical trials have shown efficacy of this compound [14,25].

Different house remedies such as application of mayonnaise or olive oil have erroneously been reported to be efficacious. In reality, these substances cause a transient period of stasis, and lice usually are not observed during a prolonged period. Interestingly, the majority of "resurrected" lice after exposure to mosquito^® ^recovered from "no vital signs" (data not shown).

Many *in vitro *studies used the inability to walk in a progressive fashion or loss of righting reflex when rolled over as criteria for mortality [9]. We believe that the more stringent criteria used in the present bioassay are adequate, if lice are observed for a period of six hours or more. In fact, mortality usually remains relatively constant after 3-6 hours of observation [13,27] and our study confirmed this finding. Other authors used "no vital signs", including complete cessation of gut peristalsis, as criterion for mortality [9].

Even in control lice mortality increased considerably after 3 - 6 hours [13], owing to dehydration. A longer observation period would considerably bias efficacies of tested products. Thus, we observed lice up to 6 hours with an additional final assessment after 24 hours to assess mortality in control group not exposed to pediculicides. Clearly, the increased "efficacy" of some products after 6 hours of observation should be interpreted with caution, due to increasing mortality in the control group.

Permethrin and malathion are generally considered safe, when used correctly, but an increased awareness that neurotoxical products may be harmful has been influencing head lice therapy in the last years [20]. In some cases these insecticides may cause adverse events such as skin irritation, paraesthesia, exacerbation of asthma and allergic reactions. Incorrect use may cause more severe adverse events. For example, a patient developed neck dystonia for 24 hours after applying 5% permethrin and not washing for 10 hours [31]. One case control study found an OR of 1.9 (95% CI: 1.1-3.3) for acute leukaemia in childhood, if insecticidal pediculicides were used in the past [32]. Clearly, further studies are needed to prove causal relationship of this association, but in general care should be taken when using neurotoxic pediculicides. In countries where safe alternatives are available with efficacy proven in well-designed studies, we recommend to avoid the use of topical neurotoxic insecticides.

## Conclusions

We have shown in a standardized bioassay that a two-phase dimeticone product is a highly efficacious product against head lice. The removal of fragrances as compared to NYDA^® ^did not affect efficacy. Permethrin showed a satisfactory efficacy, but the majority of lice treated with pyrethrum survived for a prolonged period. Highly concentrated dimeticone products can be seen as an alternative to neurotoxic pediculicides.

## Competing interests

JH has been scientific consultant to G. Pohl-Boskamp GmbH & Co. KG (Germany), the producer of NYDA^® ^and NYDA^® ^sensitiv. The company had no active role in study design, data analysis, interpretation of results or manuscript writing.

## Authors' contributions

JH: study design, conducted the study, statistical analysis, contributed to the manuscript. AA: statistical analysis, contributed to the manuscript. OL: study design, contributed to the manuscript. AM: study design, contributed to the manuscript. FAO: study design, conducted the study, statistical analysis, contributed to the manuscript. All authors read and approved the final manuscript.

## Pre-publication history

The pre-publication history for this paper can be accessed here:

http://www.biomedcentral.com/1471-5945/9/12/prepub
